# Supplementary material for: Anomalously warm weather and acute care visits in patients with multiple sclerosis: A retrospective study of privately insured individuals in the US
Source: PLoS Med. 2021 Apr 26;18(4):e1003580. doi: 10.1371/journal.pmed.1003580 (PMC8109782; doi:10.1371/journal.pmed.1003580)
Supplement: S1 Analysis Plan — (DOCX) [file pmed.1003580.s002.docx]

**S1 Analysis Plan**

The objective of the present study is to examine the implications of anomalously warm weather for individuals living with multiple sclerosis (MS), a chronic demyelinating disease of the central nervous system (CNS). Our analysis leverages extant data from the Optum Clinformatics Data Mart Database (OptumInsight, Eden Prairie, MN), a large, de-identified commercial and Medicare Advantage claims database. Patient-level commercial claims data are available from January 1, 2003 to December 31, 2017. Member enrollment data; diagnostic codes from outpatient, emergency department, and inpatient visits; and pharmacy claims are deterministically linked across file types with a unique patient identifier. The primary objectives of our statistical analyses were to:

1. Examine the association between periods of anomalously warm weather and MS-related outpatient, emergency department, and inpatient visits among individuals with MS.
2. Examine heterogeneity in these associations by sex, age, season, and region.

Because heat sensitivity is a recognized clinical feature of MS, we hypothesized that risk of MS-related healthcare visits would be increased during periods of anomalously warm weather. Below, we provide details of our analysis plan. We specify which analyses were incorporated post-hoc to further characterize the relationship between anomalously warm weather and MS-related healthcare visits, and which were incorporated in response to comments made during peer review.

Study population

Individuals eligible for the present study were between the ages of 18–64 years, resided in any of the lower 48 states or District of Columbia, were eligible for insurance for at least one month, and were identified as having MS based on a previously validated algorithm.[1] This algorithm requires at least three MS-related claims for any combination of inpatient, outpatient, or disease-modifying therapy (DMT) claims within 365 days. MS-related inpatient and outpatient claims are identified using primary diagnostic code 340 from the *International Classification of Diseases, Ninth Revision* (*ICD-9*), and G35 from the *ICD-10*. Prescription claims for DMT include interferon beta-1a-SC, interferon beta-1a-IM, interferon beta-1b-SC, pegylated interferon beta 1b, glatiramer acetate, dimethyl fumarate, fingolimod, siponimod, teriflunomide, cladribine, mitoxantrone, alemtuzumab, ocrelizumab, or natalizumab.

Exposure – Anomalously Warm Weather

The primary exposure of interest for the present study is anomalously warm weather, which will be defined using temperature data at the county-month level. Temperature data are available through the European Centre for Medium-Range Weather Forecast’s (ECMWF) open-access ERA5 reanalysis.[2] We will define anomalies as a deviation of monthly average temperatures from the long-term average temperature over the study period for each calendar month by at least 1.5˚C. Alternative metrics will include a continuous measure of the deviation between monthly average and long-term average temperatures, continuous, monthly average temperature and a measure of cool measure anomalies in which the monthly average temperatures is at least 1.5˚C below the long-term average for that calendar month. We will also create a series of alternative exposure measured in which we varied the threshold at which we defined anomalously warm weather (0.5, 1.0, 2.0˚C above the long-term average).

*Modifications carried out during peer review*

In response to comments made during peer review, we used the ECMWF temperature data to create several alternative exposure metrics. These included:

- A version of the main exposure variable in which the anomalies were lagged forward one month.
- A version of the main exposure variable in which the anomalies were lagged backward one month.
- A categorical variable for the deviation between monthly average and long-term average temperature, in which the categories were defined as ≥ 0.0˚C, > 0.0 and ≤1.5˚C, >1.5 and ≤3.0˚C, >3.0 and ≤ 4.5˚C and >4.5˚C.
- A continuous measure of the number of days each month where the temperature exceeded the long-term average for that calendar day.
- A categorical measure of the number of days each month where the temperature exceeded the long-term average for that calendar day, with categories defined as 1 – 6; 7 – 9; 10 – 13; 14 or more anomalous days with zero anomalous days as the referent groups.

Outcome – MS-related healthcare visits

We will identify MS-related outpatient, emergency department, and inpatient visits using diagnostic code 340 (*ICD-9*) and G35 (*ICD-10*) in any diagnostic position. We will then create an indicator variable for each visit type that equals one for any month with at least one MS-related outpatient, emergency, or inpatient visit – respectively – and equals zero otherwise. We will create an indicator variable for visits unrelated to MS which equals one for outpatient, emergency, or inpatient visit in which the diagnostic codes 340 and G35 areabsent and zero otherwise.

*Modifications carried out during peer review*

We modified our outcome definition to include only MS-related healthcare visits to only the first, second, and third diagnostic position.

Covariates

We will define sex (women, men), categorical age (18–25, 26–35; 36–45; 46–55, 56–64 years), and calendar year using eligibility files. We will define time-varying county, state, and region of residence using five-digit zip codes and season for each respondent.

*Modifications carried out during peer review*

In response to requests from reviewers, we defined age as a continuous rather than categorical variable in the analyses described below, and assigned climate zones based on county of residence using data from the U.S. Department of Energy.[3]

Statistical Analysis

We will conduct an individual-level, repeated measures analysis. For our primary analysis, we will use generalized linear models with the binomial family and log link to estimate risk ratios (RR) for the association between anomalously warm weather and MS-related outpatient, emergency department, and inpatient visits, respectively. We will adjust for *a priori* specified covariates that could potentially confound the association of interest. These include sex, categorical age, and fixed effects for state and calendar year. For all analyses, we will calculate robust standard errors to account for repeated measures within individuals and potential non-independence of outcomes within counties. We will use fitted models to estimate associations on the additive scale. Because of sex differences in MS incidence, response to treatment, and healthcare utilization,[4-8] we repeated our primary analyses stratified by sex.

Secondary analyses will be conducted within subgroups defined by age category, region, season, and by region within season. We will also examine the association between anomalously cool weather and MS-related healthcare visits. Sensitivity analyses will include an examination of the association between the continuous difference between monthly average and long-term average temperatures and MS-related healthcare visits; the association between average monthly temperature and MS-related healthcare visits; and an analysis using alternative thresholds to define anomalies (0.5, 1.0, 2.0˚C).

All statistical analyses were performed with R version 3.2.3 (R Foundation for Statistical Computing, Vienna, Austria).

*Modifications carried out during peer review*

We made several revisions to our statistical analyses in response to comments made during peer review, which are detailed below:

- We defined age as continuous with natural splines rather than as a categorical variable in all models.
- Secondary analyses
  - We included a subgroup analysis by climate zone.
- Formal test of interaction
  - We conducted formal tests of interaction using a p-value for interaction between our exposure variable and sex, age group, season, region, and climate zone.
- Sensitivity analyses
  - We further characterized the importance of the magnitude of the deviation of monthly average temperatures from long-term average temperatures by (1) repeating our main analysis using a categorical variable for the difference between monthly average and long-term average temperatures, and (2) defining an alternative, categorical metric for the number of anomalously warm days per month as a categorical variable.
  - We lagged the exposure metric forward and backward by one month and then repeated our main analysis.
- Negative exposure controls
  - Because our negative outcome control analysis still showed persistent – albeit attenuated – associations, we included a new negative exposure controls analysis in which we repeated our main analysis after randomly permuting the exposure variable.
  - Because cold sensitivity is not a clinical feature of multiple sclerosis, we feel that our analysis of anomalously cool weather – which we initially presented as a secondary analysis – is more appropriate as a negative exposure control analysis.

**References**

1. Culpepper, W.J., et al., *Validation of an algorithm for identifying MS cases in administrative health claims datasets.* Neurology, 2019. **92**(10): p. e1016-e1028.

2. ECMWF. *ERA5*. 2020 [cited 2020 April 10]; Available from: <https://www.ecmwf.int/en/forecasts/datasets/reanalysis-datasets/era5>.

3. Baechler, M.C., et al., *Guide to determining climate regions by county.* Pacific Northwest National Laboratory & Oak Ridge National Laboratory, 2010. **7**: p. 1-34.

4. Koch-Henriksen, N., et al., *Incidence of MS has increased markedly over six decades in Denmark particularly with late onset and in women.* Neurology, 2018. **90**(22): p. e1954-e1963.

5. Alonso, A. and M.A. Hernán, *Temporal trends in the incidence of multiple sclerosis: a systematic review.* Neurology, 2008. **71**(2): p. 129-135.

6. Magyari, M., et al., *Gender effects on treatment response to interferon‐beta in multiple sclerosis.* Acta Neurologica Scandinavica, 2014. **130**(6): p. 374-379.

7. Manteuffel, M., et al., *Influence of patient sex and gender on medication use, adherence, and prescribing alignment with guidelines.* Journal of women's health, 2014. **23**(2): p. 112-119.

8. Owens, G., *Gender differences in health care expenditures, resource utilization, and quality of care.* Journal of Managed Care Pharmacy, 2008. **14**(3): p. 2-6.
