# Supplementary figures and images for: Anomalously warm weather and acute care visits in patients with multiple sclerosis: A retrospective study of privately insured individuals in the US
Source: PLoS Med. 2021 Apr 26;18(4):e1003580. doi: 10.1371/journal.pmed.1003580 (PMC8109782; doi:10.1371/journal.pmed.1003580)

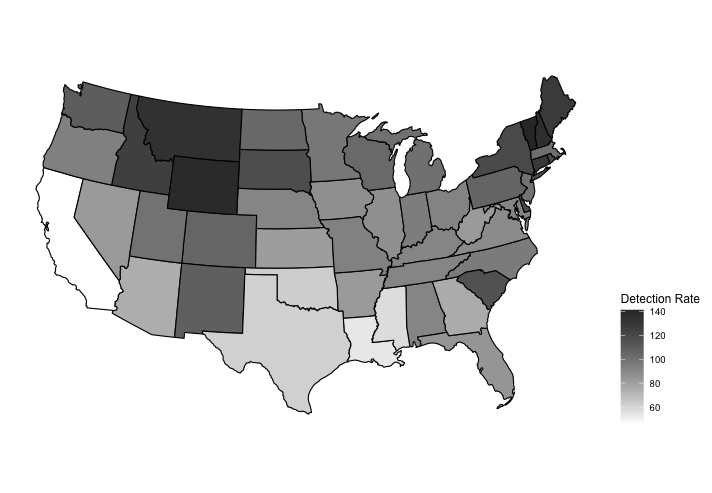

Supplement: S1 Fig — Shading intensity represents the number of MS cases detected per 100,000 person-months of follow-up in the Optum database by state from 2003 to 2017. Patients with MS were identified from the Optum’s Clinformatics Data Mart database using a previously validated algorithm, which required at least 3 MS-related inpatient, outpatient, or DMT claims within a 365-day period. MS-related claims were identified using primary diagnostic code 340 from the ICD-9 and G35 from the ICD-10. Prescription claims for DMT were identified as any dispensation for interferon beta-1a-SC, interferon beta-1a-IM, interferon beta-1b-SC, pegylated interferon beta 1b, glatiramer acetate, dimethyl fumarate, fingolimod, siponimod, teriflunomide, cladribine, mitoxantrone, alemtuzumab, ocrelizumab, or natalizumab. Maps were produced using publicly available US Census base maps accessed via the “usmaps” package for R Statistical Software v 4.0.0. DMT, disease-modifying therapy; ICD, International Classification of Diseases; MS, multiple sclerosis. (TIF) [file pmed.1003580.s003.tif]

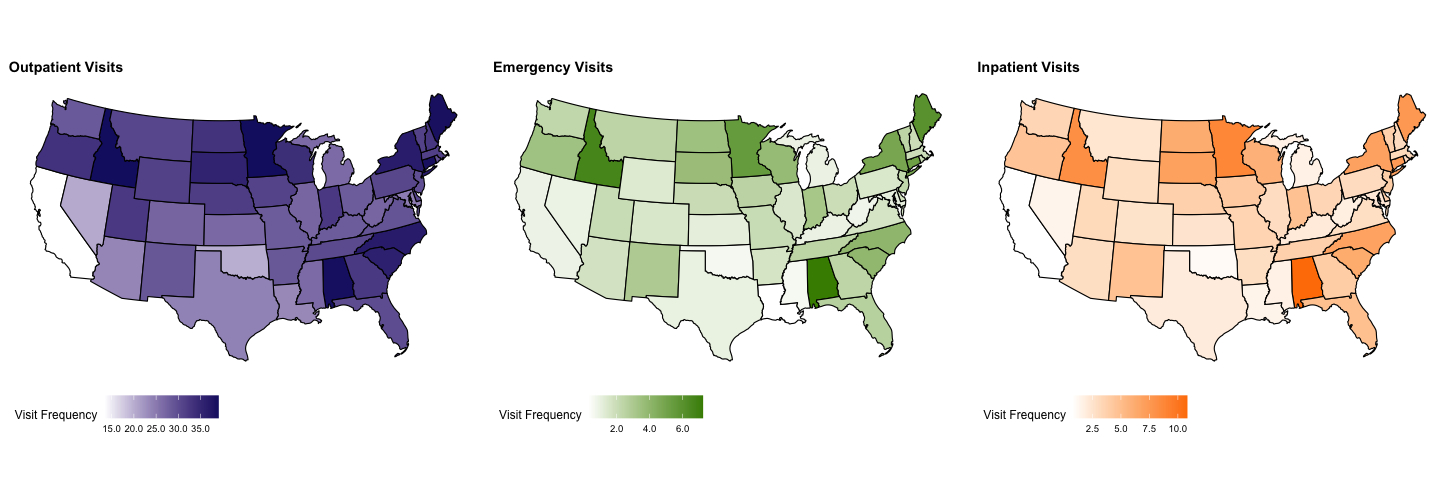

Supplement: S2 Fig — Shading intensity represents the percentage of person-months in which at least 1 MS-related outpatient, emergency department, or inpatient visit, respectively, for each state from 2003 to 2017 among the 106,225 patients with MS identified in the Clinformatics Data Mart Database. MS-related inpatient and outpatient claims were identified using primary diagnostic code 340 from the ICD-9 and G35 from the ICD-10. Maps were produced using publicly available US Census base maps accessed via the “usmaps” package for R Statistical Software v 4.0.0. ICD, International Classification of Diseases; MS, multiple sclerosis. (TIF) [file pmed.1003580.s004.tif]

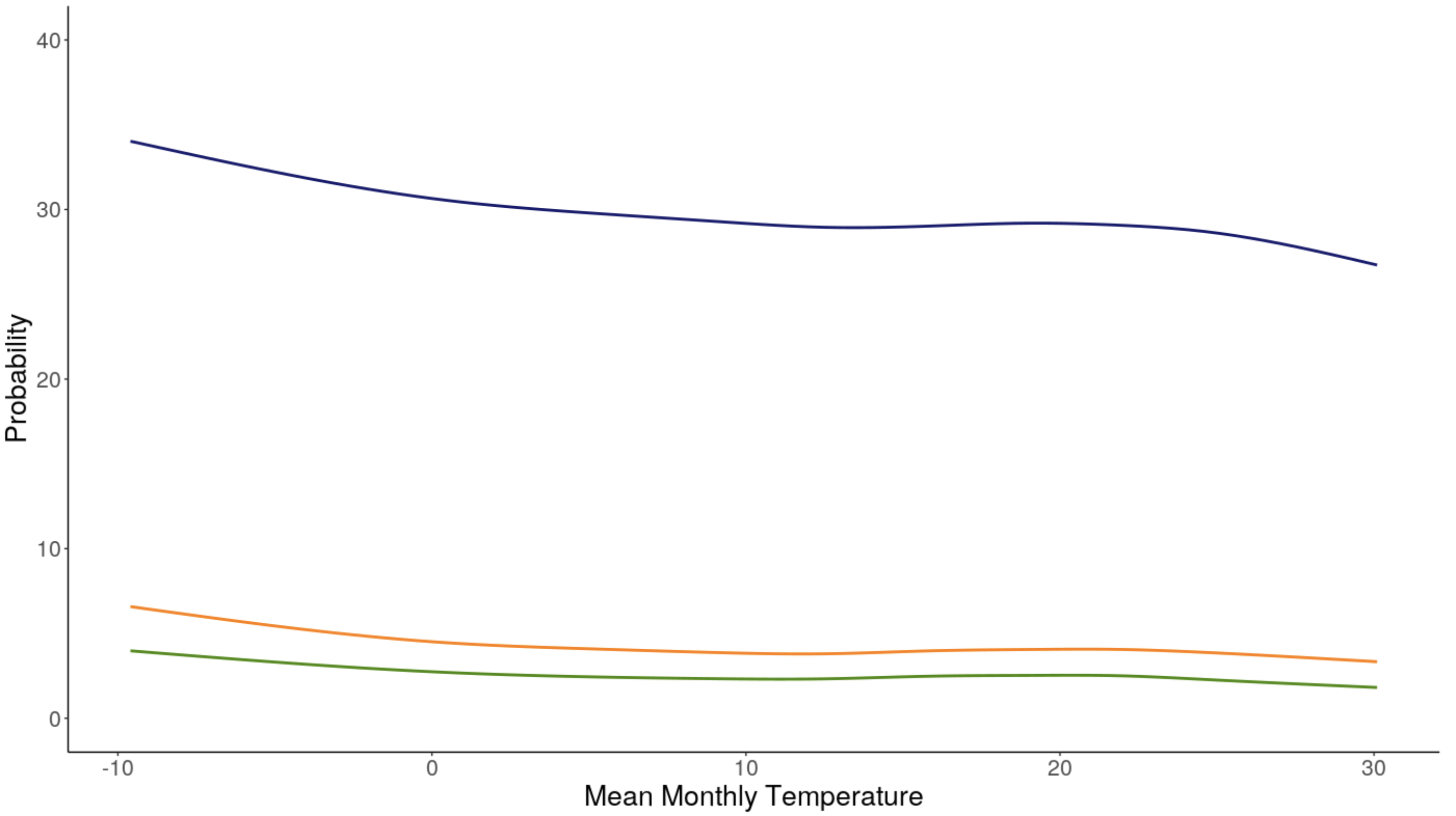

Supplement: S3 Fig — The above figure depicts the predicted probability of MS-related outpatient, emergency department, and inpatient visits at mean monthly temperatures between −10 and 30°C. We used generalized linear models with the binomial family and log link specified. We captured potential nonlinearities in the exposure–response with mean monthly temperatures using natural splines with 3 degrees of freedom. All models were adjusted for continuous age with a natural spline and sex (male or female), and we included a set of fixed effects for calendar year and state. MS, multiple sclerosis. (TIF) [file pmed.1003580.s005.tif]
