# Supplementary material for: Anomalously warm weather and acute care visits in patients with multiple sclerosis: A retrospective study of privately insured individuals in the US
Source: PLoS Med. 2021 Apr 26;18(4):e1003580. doi: 10.1371/journal.pmed.1003580 (PMC8109782; doi:10.1371/journal.pmed.1003580)
Supplement: S1 Table — (DOCX) [file pmed.1003580.s006.docx]

**S1 Table. Temperature variation (˚C) by region and season, 2003–2017^1,2^**

|  | Minimum | 1^st^ Quartile | Median | 3^rd^ Quartile | Maximum |
| --- | --- | --- | --- | --- | --- |
| **Overall** | | | | | |
| Midwest | -20.6 | 1.13 | 11.0 | 19.4 | 31.3 |
| Northeast | -17.1 | 0.5 | 9.2 | 17.7 | 25.4 |
| South | -16.7 | 9.3 | 16.7 | 23.2 | 33.8 |
| West | -14.5 | 4.2 | 10.7 | 10.5 | 17.1 |
| **Winter** | | | | | |
| Midwest | -20.6 | -7.4 | -3.4 | -0.4 | 9.4 |
| Northeast | -17.1 | -5.6 | -3.0 | -0.6 | 8.4 |
| South | -16.7 | 2.2 | 5.7 | 9.4 | 24.7 |
| West | -14.5 | -3.1 | 1.1 | 5.9 | 17.2 |
| **Spring** | | | | | |
| Midwest | -11.2 | 5.2 | 10.3 | 14.4 | 23.6 |
| Northeast | -10.3 | 2.7 | 7.8 | 12.4 | 18.0 |
| South | -8.9 | 11.9 | 16.1 | 19.7 | 29.0 |
| West | -8.5 | 5.4 | 9.1 | 12.7 | 27.9 |
| **Summer** | | | | | |
| Midwest | 13.0 | 20.4 | 21.9 | 23.6 | 31.4 |
| Northeast | 13.7 | 18.6 | 20.1 | 21.7 | 25.4 |
| South | 14.8 | 23.5 | 25.7 | 27.4 | 33.9 |
| West | 7.9 | 16.9 | 19.7 | 22.7 | 35.2 |
| **Fall** | | | | | |
| Midwest | -9.0 | 6.4 | 11.2 | 16.7 | 24.7 |
| Northeast | -1.6 | 5.2 | 10.5 | 15.6 | 21.2 |
| South | -1.4 | 12.1 | 16.8 | 21.4 | 29.9 |
| West | -6.6 | 6.2 | 11.4 | 16.1 | 31.1 |

1. We defined U.S. Census Regions as the **Northeast** (Connecticut, Massachusetts, Maine, New Hampshire, New Jersey, New York, Pennsylvania, Rhode Island, Vermont,); the **South** (Alabama, Arkansas, Delaware, District of Columbia, Florida, Georgia, Kentucky, Louisiana, Maryland, Mississippi, North Carolina, Oklahoma, South Carolina, Tennessee, Texas, Virginia, and West Virginia); the **Midwest** (Illinois, Indiana, Iowa, Kansas, Michigan, Minnesota, Missouri, Nebraska, North Dakota, Ohio, South Dakota, Wisconsin); and the **West** (Arizona, California, Colorado, Idaho, Montana, Nevada, New Mexico, Oregon, Washington, and Wyoming).
2. We defined winter as December – February; spring as March – May; Summer as June – August; and Fall as September – November.
