# Supplementary material for: Anomalously warm weather and acute care visits in patients with multiple sclerosis: A retrospective study of privately insured individuals in the US
Source: PLoS Med. 2021 Apr 26;18(4):e1003580. doi: 10.1371/journal.pmed.1003580 (PMC8109782; doi:10.1371/journal.pmed.1003580)
Supplement: S3 Table — MS, multiple sclerosis. (DOCX) [file pmed.1003580.s008.docx]

**S3 Table. Excess MS-related visits and anomalously warm weather, 2003–2007^1,2^**

|  | **Risk Difference ^3^**  (95% CI) **^5^** | **Excess Visits ^4^**  (95% CI) **^5^** |
| --- | --- | --- |
| **Outpatient Visits** | 0.0005 (0.0003–0.0007) | 1,960 (1,126–2,794) |
| **Emergency Visits** | 0.0002 (0.0001–0.0002) | 592 (305–878) |
| **Inpatient Visits** | 0.0003 (0.0002–0.0004) | 1,260 (892–1,628) |

1. We defined anomalously warm weather as any month in which the average temperature was at least 1.5˚C above the long-term average for that month and county
2. We defined MS-related visits as those with diagnostic codes 340 (ICD-9) and G35 (ICD-10) for the first, second, or third diagnostic position.
3. We used fitted log-linear models to predict the probability of each type of MS-related visit under the observed distribution of warm weather anomalies and if there had been zero warm weather anomalies. We calculated the risk difference as the difference between the predicted probability under the observed distribution and predicted probability had no warm weather anomalies occurred. All models were adjusted for continuous age defined using natural splines, sex (male, female), and included a set of indicator variables to control for confounding by state of residence and calendar year.
4. To calculate the excess visits associated with warm weather anomalies, we multiplied the predicted probability under the observed distribution of warm weather anomalies by the total duration of follow-up in person-months and multiplied the predicted probability under the counterfactual scenario in which no warm weather anomalies occurred and calculated the difference. This estimate of the excess visits constitutes a lower bound in that it is based on the assumption that an individual has no more than one outpatient, emergency department, or inpatient visit in a given calendar month.
5. We estimated 95% confidence intervals using a bootstrap procedure with 1,000 repetiti
