# Supplementary material for: Anomalously warm weather and acute care visits in patients with multiple sclerosis: A retrospective study of privately insured individuals in the US
Source: PLoS Med. 2021 Apr 26;18(4):e1003580. doi: 10.1371/journal.pmed.1003580 (PMC8109782; doi:10.1371/journal.pmed.1003580)
Supplement: S4 Table — MS, multiple sclerosis. (DOCX) [file pmed.1003580.s009.docx]

**S4 Table. Anomalously warm weather and MS-related visits by age category, 2003–2017 ^1,2,3,4^**

|  | **Age 18 – 25**  RR (95% CI) | **Age 26 – 35**  RR (95% CI) | **Age 36 – 45**  RR (95% CI) | **Age 46 – 55**  RR (95% CI) | **Age 56 – 64**  RR (95% CI) |
| --- | --- | --- | --- | --- | --- |
| **Outpatient Visits ^5^** | 1.003 (0.968 – 1.039) | 1.001 (0.987 – 1.015) | 1.010 (1.000 – 1.019) | 1.011 (1.003 – 1.019) | 1.013 (1.005 – 1.021) |
| **Emergency Visits** | 1.009 (0.841 – 1.212) | 1.044 (0.974 – 1.118) | 1.042 (0.996 – 1.091) | 1.012 (0.982 – 1.042) | 1.043 (1.015 – 1.073) |
| **Inpatient Visits** | 0.992 (0.871 – 1.130) | 0.972 (0.920 – 1.027) | 1.032 (0.999 – 1.065) | 1.048 (1.020 – 1.076) | 1.053 (1.031 – 1.073) |

1. We defined anomalously warm weather as any month in which the average temperature was at least 1.5˚C above the long-term average for that month and county
2. We defined MS-related visits as those with diagnostic codes 340 (ICD-9) and G35 (ICD-10) for the first, second, or third diagnostic position.
3. We used generalized log-linear models to estimate risk ratios within strata defined by age category (18 – 25; 26 – 35; 36 – 45; 46 – 55; 56 – 64). All models were adjusted for sex (male, female), and included a set of indicator variables to control for confounding by state of residence and calendar year. We calculated robust-standard errors to account for potential non-independence of outcomes within individuals over time and within counties.
4. We conducted a formal test for interaction using models with a product term between the exposure variable and age group. The interaction was not statistically significant in our analysis of outpatient visits for age category 26 – 35 (p=0.78), age category 36 – 45 (p=0.71), age category 46 – 55 (p=0.49), or age category 56 – 64 (p=0.15); the interaction was not statistically significant in our analysis of emergency department visits for age category 26 – 35 (p=0.88), age category 36 – 45 (p=0.97, age category 46 – 55 (p = 0.96), or age category 56 – 64 (p=0.73); the interaction was not statistically significant in our analysis of inpatient visits for age category 26 – 35 (p = 0.75), age category 36 – 45 (p = 0.28), age category 46 – 55 (p = 0.13), or age category 56-64 (p = 0.12) with age category 18 – 25 as the referent category.
5. Included visits to medical offices, outpatient hospitals, urgent care facilities, independent clinics, walk-in retail health clinics, and state or local public health clinics.
