# Supplementary material for: Anomalously warm weather and acute care visits in patients with multiple sclerosis: A retrospective study of privately insured individuals in the US
Source: PLoS Med. 2021 Apr 26;18(4):e1003580. doi: 10.1371/journal.pmed.1003580 (PMC8109782; doi:10.1371/journal.pmed.1003580)
Supplement: S5 Table — MS, multiple sclerosis. (DOCX) [file pmed.1003580.s010.docx]

**S5 Table. Anomalously warm weather and MS-related visits by season, 2003–2017 ^1,2,3,4,5^**

|  | **Winter**  RR (95% CI) | **Spring**  RR (95% CI) | **Summer**  RR (95% CI) | **Fall**  RR (95% CI) |
| --- | --- | --- | --- | --- |
| **Outpatient Visits ^6^** | 1.025 (1.015 – 1.034) | 0.998 (0.988 – 1.008) | 1.016 (1.003 – 1.029) | 1.008 (0.998 – 1.018) |
| **Emergency Visits** | 1.021 (0.984 – 1.060) | 1.016 (0.983 – 1.049) | 1.030 (0.978 – 1.085) | 1.014 (0.972 – 1.057) |
| **Inpatient Visits** | 1.037 (1.005 – 1.071) | 1.016 (0.988 – 1.044) | 1.008 (0.967 – 1.050) | 1.004 (0.973 – 1.036) |

1. We defined anomalously warm weather at the county level as any month in which the average temperature was > 1·5˚C above the long-term average for that month
2. We defined MS-related visits as those with diagnostic codes 340 (ICD-9) and G35 (ICD-10) for the first, second, or third diagnostic position.
3. We defined winter as December – February; spring as March – May; Summer as June – August; and Fall as September – November.
4. We used generalized linear models with the binomial family and log link specified to estimate risk ratios. All models included controls categorical sex (male, female), continuous age defined by natural splines with three degrees of freedom, and a set of indicator variables for state and calendar year. We calculated robust-standard errors to account for potential non-independence of outcomes within individuals over time and within counties.
5. We conducted a formal test for interaction using models with a product term between the exposure variable and season. The interaction was not statistically significant in our analysis of outpatient visits for the winter (p=0.03) but not for the spring (p=0.14) or summer (p =0.35); The interaction was not statistically significant in our analysis of emergency department visits for the winter (p=0.97), spring (p=0.80), or summer (p=0.58); The interaction was not statistically significant in our analysis of inpatient visits for the winter (p=0.19), spring (p=0.56), or summer (p=0.99) with fall as the referent season.
6. Included visits to medical offices, outpatient hospitals, urgent care facilities, independent clinics, walk-in retail health clinics, and state or local public health clinics.
