# Supplementary material for: Anomalously warm weather and acute care visits in patients with multiple sclerosis: A retrospective study of privately insured individuals in the US
Source: PLoS Med. 2021 Apr 26;18(4):e1003580. doi: 10.1371/journal.pmed.1003580 (PMC8109782; doi:10.1371/journal.pmed.1003580)
Supplement: S6 Table — MS, multiple sclerosis. (DOCX) [file pmed.1003580.s011.docx]

**S6 Table. Anomalously warm weather and MS-related visits by U.S. Census Region, 2003–2017** **^1,2,3,4,5^**

|  | **Midwest**  RR (95% CI) **^3^** | **Northeast**  RR (95% CI) **^3^** | **South**  RR (95% CI) **^3^** | **West**  RR (95% CI) **^3^** |
| --- | --- | --- | --- | --- |
| **Outpatient Visits ^6^** | 1.000 (0.992 – 1.007) | 1.011 (0·995 – 1.027) | 1.024 (1.017 – 1.032) | 0.987 (0.976 – 0.999) |
| **Emergency Visits** | 1.024 (0.988 – 1.061) | 1.009 (0.962 – 1.059) | 1.072 (1.042 – 1.102) | 0.989 (0.941 – 1.039) |
| **Inpatient Visits** | 1.014 (0.989 – 1.039) | 1.066 (1.021 – 1.114) | 1.084 (1.059 – 1.110) | - 1. .915 – 1.007) |

1. We defined anomalously warm weather as any month in which the average temperature was at least 1.5˚C above the long-term average for that month and county
2. We defined MS-related visits as those with diagnostic codes 340 (ICD-9) and G35 (ICD-10) for the first, second, or third diagnostic position.
3. We defined U.S. Census Regions as the **Northeast** (Connecticut, Massachusetts, Maine, New Hampshire, New Jersey, New York, Pennsylvania, Rhode Island, Vermont,); the **South** (Alabama, Arkansas, Delaware, District of Columbia, Florida, Georgia, Kentucky, Louisiana, Maryland, Mississippi, North Carolina, Oklahoma, South Carolina, Tennessee, Texas, Virginia, and West Virginia); the **Midwest** (Illinois, Indiana, Iowa, Kansas, Michigan, Minnesota, Missouri, Nebraska, North Dakota, Ohio, South Dakota, Wisconsin); and the **West** (Arizona, California, Colorado, Idaho, Montana, Nevada, New Mexico, Oregon, Washington, and Wyoming).
4. We used generalized linear models with the binomial family and log link specified to estimate risk ratios. All models included controls categorical sex (male, female), continuous age defined by natural splines with three degrees of freedom, and a set of indicator variables for state and calendar year. We calculated robust-standard errors to account for potential non-independence of outcomes within individuals over time and within counties.
5. We conducted a formal test for interaction using models with a product term between the exposure variable and region. In our analysis of outpatient visits, the interaction was statistically significant for the Northeast (p=0.002); the South (p<0.001); and the West (p <0.001); in our analysis of emergency department visits, the interaction was not statistically significant for the Northeast (p=0.14) but was significant for the South (p<0.001) and the West (p=0.01); and in our analysis of inpatient visits, the interaction was statistically significant for the Northeast (p<0.001), South (p<0.001), and West (p=0.02) with the Midwest as the referent category.
6. Included visits to medical offices, outpatient hospitals, urgent care facilities, independent clinics, walk-in retail health clinics, and state or local public health clinics
