# Supplementary material for: Anomalously warm weather and acute care visits in patients with multiple sclerosis: A retrospective study of privately insured individuals in the US
Source: PLoS Med. 2021 Apr 26;18(4):e1003580. doi: 10.1371/journal.pmed.1003580 (PMC8109782; doi:10.1371/journal.pmed.1003580)
Supplement: S7 Table — MS, multiple sclerosis. (DOCX) [file pmed.1003580.s012.docx]

**S7 Table. Anomalously warm weather and MS-related visits by region and season, 2003–2017 ^1,2,3,4,5^**

|  | **Winter**  RR (95% CI) | **Spring**  RR (95% CI) | **Summer**  RR (95% CI) | **Fall**  RR (95% CI) |
| --- | --- | --- | --- | --- |
| **MIDWEST** | | | | |
| **Outpatient Visits ^6^** | 0.991 (0.976 – 1·006) | 0.977 (0.962 – 0.991) | 0.992 (0.975 – 1.009) | 1.026 (1·011 – 1·041) |
| **Emergency Visits** | 0.974 (0.917 – 1.035) | 1.031 (0.978 – 1.086) | 0.998 (0.939 – 1.062) | 1.095 (1.032 – 1.161) |
| **Inpatient Visits** | 0.953 (0.912 – 0.997) | 1.018 (0.973 – 1.065) | 0.954 (0.904 – 1.005) | 1.022 (0.975 – 1.071) |
| **NORTHEAST** | | | | |
| **Outpatient Visits ^6^** | 1.027 (0.988 – 1.067) | 0.992 (0.952 – 1.034) | 1.022 (0.964 – 1.083) | 1.018 (0.988 – 1.050) |
| **Emergency Visits** | 1.011 (0.880 – 1.162) | 0.987 (0.853 – 1.141) | 0.960 (0.751 – 1.228) | 1.132 (0.967 – 1.326) |
| **Inpatient Visits** | 0.941 (0.845 – 1.048) | 1.094 (0.950 – 1.260) | 0·976 (0.798 – 1.195) | 1.022 (0.906 – 1.152) |
| **SOUTH** | | | | |
| **Outpatient Visits ^6^** | 1.044 (1.030 – 1.058) | 1.014 (0.999 – 1.030) | 1.059 (1.039 – 1.079) | 1.013 (0.997 – 1.028) |
| **Emergency Visits** | 1.096 (1·035 – 1·160) | 1.033 (0.988 – 1.080) | 1.027 (0.934 – 1.129) | 1.034 (0.969 – 1.103) |
| **Inpatient Visits** | 1.105 (1.060 – 1.153) | 1.032 (0.997 – 1.068) | 1.054 (0.984 – 1.130) | 1.045 (0.994 – 1.098) |
| **WEST** | | | | |
| **Outpatient Visits ^6^** | 0.970 (0.940 – 1.001) | 1.016 (0.985 – 1.048) | 0.979 (0.942 – 1.017) | 0.982 (0.960 – 1.005) |
| **Emergency Visits** | 0.892 (0.807 – 0.985) | 0.965 (0.867 – 1.074) | 1.076 (0.903 – 1.281) | 1.037 (0.931 – 1.156) |
| **Inpatient Visits** | 0.960 (0.871 – 1.058) | 0.927 (0.831 – 1.035) | 0.952 (0.797 – 1.138) | 0.950 (0.873 – 1.034) |

1. We defined anomalously warm weather at the county level as any month in which the average temperature was > 1·5˚C above the long-term average for that month
2. We defined MS-related visits as those with diagnostic codes 340 (ICD-9) and G35 (ICD-10) for the first, second, or third diagnostic position.
3. We defined winter as December – February; spring as Marc h – May; Summer as June – August; and Fall as September – November.
4. We defined U.S. Census Regions as the **Northeast** (Connecticut, Massachusetts, Maine, New Hampshire, New Jersey, New York, Pennsylvania, Rhode Island, Vermont,); the **South** (Alabama, Arkansas, Delaware, District of Columbia, Florida, Georgia, Kentucky, Louisiana, Maryland, Mississippi, North Carolina, Oklahoma, South Carolina, Tennessee, Texas, Virginia, and West Virginia); the **Midwest** (Illinois, Indiana, Iowa, Kansas, Michigan, Minnesota, Missouri, Nebraska, North Dakota, Ohio, South Dakota, Wisconsion); and the **West** (Arizona, California, Colorado, Idaho, Montana, Nevada, New Mexico, Oregon, Washington, and Wyoming).
5. We used generalized log-linear models to estimate risk ratios by U.S. Census Region and season. All models included controls categorical sex (male, female), continuous age defined by natural splines with three degrees of freedom, and a set of indicator variables for state and calendar year. We calculated robust-standard errors to account for potential non-independence of outcomes within individuals over time and within counties.
6. Included visits to medical offices, outpatient hospitals, urgent care facilities, independent clinics, walk-in retail health clinics, and state or local public health clinics.
