# Supplementary material for: Anomalously warm weather and acute care visits in patients with multiple sclerosis: A retrospective study of privately insured individuals in the US
Source: PLoS Med. 2021 Apr 26;18(4):e1003580. doi: 10.1371/journal.pmed.1003580 (PMC8109782; doi:10.1371/journal.pmed.1003580)
Supplement: S8 Table — MS, multiple sclerosis. (DOCX) [file pmed.1003580.s013.docx]

**S8 Table. Anomalously warm weather and MS-related visits by climate zone, 2003–2017** **^1,2,3,4,5^**

|  | **Outpatient visits ^6^**  RR (95% CI) | **Emergency Visits**  RR (95% CI) | **Inpatient Visits**  RR (95% CI) |
| --- | --- | --- | --- |
| **Marine** | 1.006 (0.968 – 1.046) | 0.949 (0.876 – 1.028) | 0.961 (0.851 – 1.085) |
| **Very Cold** | 1.011 (0.985 – 1.038) | 1.026 (0.922 – 1.142) | 1.068 (0.977 – 1.167) |
| **Cold** | 1.003 (0.996 – 1.011) | 1.026 (0.995 – 1.058) | 1.029 (1.003 – 1.056) |
| **Mixed-Humid** | 1.012 (1.004 – 1.020) | 1.044 (1.011 – 1.077) | 1.064 (1.039 – 1.090) |
| **Mixed-Dry** | 0.965 (0.920 – 1.011) | 0.939 (0.810 – 1.089) | 1.025 (0.965 – 1.089) |
| **Hot-Humid** | 1.034 (1.022 – 1.046) | 1.109 (1.053 – 1.168) | 1.090 (1.048 – 1.135) |
| **Hot-Dry** | 0.991 (0.962 – 1.022) | 0.973 (0.896 – 1.056) | 0.907 (0.832 – 0.988) |

1. We defined anomalously warm weather as any month in which the average temperature was at least 1.5˚C above the long-term average for that month and county
2. We defined MS-related visits as those with diagnostic codes 340 (ICD-9) and G35 (ICD-10) for the first, second, or third diagnostic position.
3. We used climate zone designations defined by the U.S. Department of Energy Building America Program.
4. We used generalized linear models with the binomial family and log link specified to estimate risk ratios. All models included controls categorical sex (male, female), continuous age defined by natural splines with three degrees of freedom, and a set of indicator variables for state and calendar year. We calculated robust-standard errors to account for potential non-independence of outcomes within individuals over time and within counties.
5. We conducted a formal test for interaction using models with a product term between the exposure variable and region adjusted for the above specified covariates. In our analysis of outpatient visits, the interaction was statistically significant for mixed-humid (p=0.02), hot-humid (p=0.04) and hot-dry (p<0.001) climates but not for very cold (p=0.27) or mixed-dry (p=0.53) climates; in our analysis of emergency department visits the interaction was statistically significant for mixed-dry (p=0.04), hot-humid (p<0.001), hot-dry (p<0.001) and marine climates (p<0.001) but not for very cold (p=0.61) or mixed-humid climates (p=0.06); and in our analysis of inpatient visits, the interaction was statistically significant for mixed-humid (p=0.003), marine (p=0.002), hot-humid (0.003), and hot-dry (p<0.001) climates but not for mixed-dry (0.092) or very cold (p=0.16) climates with cold climates as the referent.
6. Included visits to medical offices, outpatient hospitals, urgent care facilities, independent clinics, walk-in retail health clinics, and state or local public health clinics
