# Supplementary material for: Anomalously warm weather and acute care visits in patients with multiple sclerosis: A retrospective study of privately insured individuals in the US
Source: PLoS Med. 2021 Apr 26;18(4):e1003580. doi: 10.1371/journal.pmed.1003580 (PMC8109782; doi:10.1371/journal.pmed.1003580)
Supplement: S9 Table — (DOCX) [file pmed.1003580.s014.docx]

**S9 Table. Difference between average monthly and long-term average temperatures, 2003–2017 ^1,2,3^**

|  | **Outpatient Visits ^4^**  RR (95% CI) | **Emergency Visits**  RR (95% CI) | **Inpatient Visits**  RR (95% CI) |
| --- | --- | --- | --- |
| ≤ 0.0˚C  (0.0 – 1.5˚C]  (1.5 – 3.0˚C]  (3.0 – 4.5˚C]  > 4.5˚C | 1.00  1.001 (0.996 – 1.006)  1.003 (0.997 – 1.010)  1.036 (1.022 – 1.049)  1.022 (1.012 – 1.040) | 1.00  0.985 (0.967 – 1.003)  1.015 (0.989 – 1.042)  1.050 (1.010 – 1.092)  1.056 (0.998 – 1.117) | 1.00  0.994 (0.980 – 1.008)  1.013 (0.993 – 1.033)  1.116 (1.081 – 1.153)  1.119 (1.070 – 1.171) |

1. Exposure is a categorical metric for deviation from monthly average temperature from the long-run average for each calendar month and county. The reference category includes all months in which the monthly average is equal to or less than the long-run average. The remaining categories are defined as deviations of the monthly average temperature from the long-run average by > 0.0 and ≤ 1.5˚C; > 1.5 and ≤ 3.0˚C; > 3.0 and ≤ 4.5˚C; and > 4.5˚C
2. We defined MS-related visits as those with diagnostic codes 340 (ICD-9) and G35 (ICD-10) for the first, second, or third diagnostic position.
3. We used generalized linear models with the binomial family and log link specified to estimate risk ratios. All models included controls categorical sex (male, female), continuous age defined by natural splines with three degrees of freedom, and a set of indicator variables for state and calendar year. We calculated robust-standard errors to account for potential non-independence of outcomes within individuals over time and within counties.
4. Visits to medical offices, outpatient hospitals, urgent care facilities, independent clinics, walk-in retail health clinics, and state or local public health clinics.
