# Supplementary material for: Anomalously warm weather and acute care visits in patients with multiple sclerosis: A retrospective study of privately insured individuals in the US
Source: PLoS Med. 2021 Apr 26;18(4):e1003580. doi: 10.1371/journal.pmed.1003580 (PMC8109782; doi:10.1371/journal.pmed.1003580)
Supplement: S10 Table — MS, multiple sclerosis. (DOCX) [file pmed.1003580.s015.docx]

**S10 Table. Number of anomalously warm days per month and MS-related visits, 2003–2017 ^1,2,3^**

|  | **Outpatient Visits ^4^**  RR (95% CI) | **Emergency Visits**  RR (95% CI) | **Inpatient Visits**  RR (95% CI) |
| --- | --- | --- | --- |
| Zero  1 – 6 days  7 – 9 days  10 – 13 days  14 days or more | 1.00  1.005 (0.989 – 1.022)  1.014 (0.997 – 1.031)  1.015 (0.998 – 1.032)  1.017 (1.000 – 1.034) | 1.00  1.034 (0.976 – 1.096)  1.028 (0.970 – 1.089)  1.009 (0.949 – 1.071)  1.052 (0.990 – 1.117) | 1.00  1.024 (0.979 – 1.070)  1.024 (0.981 – 1.069)  1.036 (0.992 – 1.082)  1.070 (1.024 – 1.117) |

1. We created an alternative exposure metric that represents the number of anomalously warm days per month. We calculated daily anomalies based on the deviation between each day between January 1, 2003 – December 31, 2017 and the corresponding average for that calendar day over the study period. We defined any daily anomaly as a deviation of at least 1.5˚C above the long-term average for that calendar day over the study period. We then created categories based on the number of anomalously warm days per month. Cutpoints correspond to the first quartile, median, and third quartile of anomalous days per month with zero anomalies as the referent.
2. We defined MS-related visits as those with diagnostic codes 340 (ICD-9) and G35 (ICD-10) for the first, second, or third diagnostic position.
3. We used generalized linear models with the binomial family and log link specified to estimate risk ratios. All models included controls categorical sex (male, female), continuous age defined by natural splines with three degrees of freedom, and a set of indicator variables for state and calendar year. We calculated robust-standard errors to account for potential non-independence of outcomes within individuals over time and within counties.
4. Included visits to medical offices, outpatient hospitals, urgent care facilities, independent clinics, walk-in retail health clinics, and state or local public health clinics.
