# Supplementary material for: Anomalously warm weather and acute care visits in patients with multiple sclerosis: A retrospective study of privately insured individuals in the US
Source: PLoS Med. 2021 Apr 26;18(4):e1003580. doi: 10.1371/journal.pmed.1003580 (PMC8109782; doi:10.1371/journal.pmed.1003580)
Supplement: S11 Table — (DOCX) [file pmed.1003580.s016.docx]

**S11 Table. Alternative thresholds for anomalously warm weather, 2003–2017 ^1,2,3^**

|  | **0.5˚ Threshold**  RR (95% CI) | **1.0˚ Threshold**  RR (95% CI) | **1.5˚ Threshold**  RR (95% CI) | **2.0˚ Threshold**  RR (95% CI) |
| --- | --- | --- | --- | --- |
| **Outpatient Visits ^4^** | 1.005 (1.001 – 1.010) | 1.007 (1.002 – 1.011) | 1.010 (1.005 – 1.015) | 1.012 (1.006 – 1.019) |
| **Emergency Visits** | 1.012 (0.995 – 1.029) | 1.018 (0.999 – 1.037) | 1.032 (1.010 – 1.054) | 1.040 (1.016 – 1.065) |
| **Inpatient Visits** | 1.031 (1.017 – 1.045) | 1.036 (1.021 – 1.051) | 1.043 (1.025 – 1.062) | 1.059 (1.037 – 1.082) |

1. We created a set of alternative indicators in which the average monthly temperature exceeded the long-term average over the study period by 0.5, 1.0, 1.5, or 2.0˚C.
2. We defined MS-related visits as those with diagnostic codes 340 (ICD-9) and G35 (ICD-10) for the first, second, or third diagnostic position.
3. We used generalized linear models with the binomial family and log link specified to estimate risk ratios. All models included controls categorical sex (male, female), continuous age defined by natural splines with three degrees of freedom, and a set of indicator variables for state and calendar year. We calculated robust-standard errors to account for potential non-independence of outcomes within individuals over time and within counties.
4. Included visits to medical offices, outpatient hospitals, urgent care facilities, independent clinics, walk-in retail health clinics, and state or local public health clinics
