# Supplementary material for: Anomalously warm weather and acute care visits in patients with multiple sclerosis: A retrospective study of privately insured individuals in the US
Source: PLoS Med. 2021 Apr 26;18(4):e1003580. doi: 10.1371/journal.pmed.1003580 (PMC8109782; doi:10.1371/journal.pmed.1003580)
Supplement: S12 Table — MS, multiple sclerosis. (DOCX) [file pmed.1003580.s017.docx]

**S12 Table. Lagged exposure variable and MS-related visits, 2003–2017 ^1,2,3,4^**

|  | **Lagged Back One Month**  RR (95% CI) | **No Lag**  RR (95% CI) | **Lagged Forward One Month**  RR (95% CI) |
| --- | --- | --- | --- |
| **Outpatient Visits ^5^** | 1.003 (0.997 – 1.009) | 1.010 (1.005 – 1.015) | 0.990 (0.985 – 0.995) |
| **Emergency Visits** | 1.015 (0.994 – 1.037) | 1.032 (1.010 – 1.054) | 1.000 (0.981 – 1.020) |
| **Inpatient Visits** | 1.028 (1.010 – 1.047) | 1.043 (1.025 – 1.062) | 1.012 (0.994 – 1.030) |

1. We defined anomalously warm weather as any month in which the average temperature was at least 1·5˚C above the long-term average for that month and county.
2. We created two alternative versions of the exposure metric that corresponds to whether there was an anomalously during the previous month (“lagged back”) and whether there was an anomaly one month in the future (“lagged forward”).
3. We defined visits unrelated to MS as any visit without diagnostic codes 340 (ICD-9) and G35 (ICD-10) in the first through 25^th^ position.
4. Log-linear models failed to converge for this analysis, therefore we used generalized linear models with the binomial family and logit link specified to estimate odds ratios. All models included controls categorical sex (male, female), continuous age defined by natural splines with three degrees of freedom, and a set of indicator variables for state and calendar year. We calculated robust-standard errors to account for potential non-independence of outcomes within individuals over time and within counties.
5. Included visits to medical offices, outpatient hospitals, urgent care facilities, independent clinics, walk-in retail health clinics, and state or local public health clinics.
