# Supplementary material for: Anomalously warm weather and acute care visits in patients with multiple sclerosis: A retrospective study of privately insured individuals in the US
Source: PLoS Med. 2021 Apr 26;18(4):e1003580. doi: 10.1371/journal.pmed.1003580 (PMC8109782; doi:10.1371/journal.pmed.1003580)
Supplement: Attachment — Submitted filename: elser.pdf [file pmed.1003580.s019.pdf]

This paper uses routine data–sets to examine the relationship between temperature anomalies and multiple sclerosis health care utilisation.

I was asked for a statistical report and I interpret that to include all aspects of the design and conduct of the study.

Points of detail

Page 6 Why model age as categorical? Categorising an essentially continuous variable wastes information (Altman and Royston, 2006; Royston et al., 2006) and leads to models which are often implausible as they predict the effect remaining flat within categories and then jumping to a new value at the category boundary. In Figure S3 the authors use a spline so why not do that for age?

Pages 6 and 7 The description of the analysis is rather brief and I do not think I could replicate it if the authors gave me the data. To be specific let me outline what I understand from the text so the authors can either confirm it or expand to dispel my confusion.

- The unit of analysis is the calendar month.
- Each month has an outcome value for each type of MS health usage. Is that binary or a count?
- If it is binary is this a log–binomial model (in R–speak family = binomial, link = log) or have the authors used what people have come to call a modified Poisson model as an approximation to the log–binomial to estimate risk ratios?
- Calendar year is presumably included as a fixed effect but is state a random effect given there are 48 of them?
- What happens if people move during their observation period? Is their location changed or are they treated as being where they started from?

Page 9 Are the sub–group differences presented here backed up by a formal test? If so it should be presented and if not then can we rely on these differences?

Page 22, Table Why are there so few from the north east?

Page 23, Figure 2 The results for women seem much less precise than I would have expected given that they represent the majority of the sample. Is there any explanation for this?

Points of more substance

I have some problems reconciling the science and the statistical analysis used here. I am certainly not an expert on the science here but let me express my doubts so the authors can consider them and if necessary rebut them.

Heat stress

On page 3 the authors state that '... heat sensitivity is a recognized feature of multiple sclerosis ...'. Their chosen exposure is heat anomalies and empirically (Figure S2) they found that in the United States these occur most often in the winter and that is when they found a relationship between exposure and outcome for at least some of their three outcomes (Table S5). Is an excess of 1.5° in winter really heat stress? At the time of writing my region is experiencing temperatures which are apparently above the seasonal average but at 14° I am not feeling any stress, unlike in the summer when they were in the high twenties, and nor do I think I am experiencing "... core temperature–induced changes..." (page 11).

There is also the issue of the failure to find any relationship between average temperature and events as exemplified in Figure S3 which is more or less flat from -3° to +27°.

Role of negative control

The authors state that the relationship between temperature anomalies and non–MS related events was attenuated. This is true but Tables S1 and S10 reveal that the relevant risk ratios were

	MS–related		MS–unrelated	
	RR	95% CI	RR	95% CI
Outpatient	1.101	1.005 to 1.015	1.000	0.995 to 1.004
Emergency	1.033	1.011 to 1.055	1.015	1.003 to 1.027
Inpatient	1.044	1.025 to 1.063	1.031	1.019 to 1.044

Table 1:

Given the considerable overlap between the intervals I am not convinced that the negative control shows what the authors hoped. Clearly something is going on here but it seems complex.

Leads and lags

Working with existing data–sets one has to take what one can find but the data available to the authors here is best suited if the relationship between anomaly and event is close in time. If heating takes a time to have its effect then events may depend more on the heat over the previous week or two and that may well have been in the previous month.

Generalisability

Will this apply to regions of the world with different climates? Specifically will it apply to a region like Europe some of which is in similar latitudes but has a different climate? There are classifications of climate and I wonder whether it might be more generalisable to use climate zones rather than the defined United States regions.

Summary

A few points for clarification and some points about the analysis strategy.

Michael Dewey

References

D G Altman and P Royston. The cost of dichotomising continuous variables. *British Medical Journal*, 332:1080, 2006.

P Royston, D G Altman, and W Sauerbrei. Dichotomizing continuous predictors in multiple regression: a bad idea. *Statistics in Medicine*, 25:127–141, 2006.
