# Supplementary material for: Anomalously warm weather and acute care visits in patients with multiple sclerosis: A retrospective study of privately insured individuals in the US
Source: PLoS Med. 2021 Apr 26;18(4):e1003580. doi: 10.1371/journal.pmed.1003580 (PMC8109782; doi:10.1371/journal.pmed.1003580)
Supplement: Attachment — Submitted filename: MS R1 - Response to Reviewers.docx [file pmed.1003580.s020.docx]

**REQUESTS FROM THE EDITORS**

* Collectively the reviewers raise some concerns around the analyses that are presented, recommend some changes in the statistical analysis approach (including how some data are coded and categorised); they also query whether the main conclusions are plausible given the data. We'd ask that these concerns are thoroughly addressed, which may include moderating some of the conclusions given those points.

We are grateful for the opportunity to revise our manuscript for ongoing consideration. We have been as responsive as possible – within the constraints of these data – and have incorporated several additional analyses as requested by the Academic Editor and Reviewers. We have also reframed the introduction, discussion, and conclusions to more clearly articulate our research question, the significance of our findings, and the limitations of our analytic approach are acknowledged. We hope the editors agree that our revised manuscript is strengthened considerably as compared with the original submission.

* Please structure your abstract using the PLOS Medicine headings (Background, Methods and Findings, Conclusions - "Methods and Findings" is a single subsection). In the last sentence of the Abstract Methods and Findings section, please include a point about any key limitation(s) of the study's methodology.

The abstract is now structured using PLOS Medicine headings with key limitations noted in the last sentence of the “Methods and Findings” section.

* Please reformat the citation style into PLOS Medicine's format (should be straight forward if using referencing software) - this should use callouts formatted as sequential numerals in square brackets (not superscript).

The references have been reformatted.

* At the moment, it's not clear that the abstract (particularly in the sentence copied below) clearly presents the main findings of the study's analysis. Specifically - "We estimated that at least 1,960 outpatient (95% CI: 1,126–2794), 592 emergency department (95% CI: 305–878), and 1,260 inpatient (95% CI: 892–1,628) MS-related visits occurred during periods of anomalously warm weather" - does this mean those numbers pertain to "additional" visits corresponding to the increased risk attributed to higher temperature? If so, that should be stated. Simply saying that those numbers of visits occur during the higher temperature periods, doesn't establish that there is a link or association. The authors could also consider including the results of primary analysis for the adjusted risk ratios attributed to higher temperatures. (In the main m/s, given as RR 1.044 (for emergency department visits, with 95%CI 1.025 - 1.063) and RR 1.033 for inpatient visits (95% CI 1.011–1.055**)).**

We agree, and our revised abstract presents the adjusted RRs from our main analysis now, stating: “Anomalously warm weather was associated with increased risk for emergency department visits (RR=1.043, 95% CI: 1.025–1.063) and inpatient visits (RR=1.032, 95% CI: 1.010–1.054). There was limited evidence of an association between anomalously warm weather and MS-related outpatient visits (RR=1.010, 95% CI: 1.005–1.015).”

* At this stage, we ask that you include a short, non-technical Author Summary of your research to make findings accessible to a wide audience that includes both scientists and non-scientists. The Author Summary should immediately follow the Abstract in your revised manuscript. This text is subject to editorial change and should be distinct from the scientific abstract. Please see our author guidelines for more information: <https://journals.plos.org/plosmedicine/s/revising-your-manuscript#loc-author-summary>

We have included a non-technical Author Summary with the revised manuscript.

* Please ensure that the study is reported according to the STROBE guideline (<http://www.equator-network.org/reporting-guidelines/strobe/>), and include the completed STROBE checklist as Supporting Information.  Please add the following statement, or similar, to the Methods: "This study is reported as per the Strengthening the Reporting of Observational Studies in Epidemiology (STROBE) guideline (S1 Checklist)."  When completing the checklist, please use section and paragraph numbers, rather than page numbers.

We have included a completed STROBE checklist with the resubmission.

* We'd ask the authors to clarify if the main analytical approach reported here corresponds to one laid out in a prospective protocol or analysis plan. Please state this (either way) early in the Methods section.

a) If a prospective analysis plan (from your funding proposal, IRB or other ethics committee submission, study protocol, or other planning document written before analyzing the data) was used in designing the study, please include the relevant prospectively written document with your revised manuscript as a Supporting Information file to be published alongside your study, and cite it in the Methods section. A legend for this file should be included at the end of your manuscript. 

b) If no such document exists, please make sure that the Methods section transparently describes when analyses were planned, and when/why any data-driven changes to analyses took place.  

c) In either case, changes in the analysis-- including those made in response to peer review comments-- should be identified as such in the Methods section of the paper, with rationale.

The main analytical approach reported here was developed prospectively in April of 2020. We have included an analysis plan with this revision that describes our originally planned analyses and details the changes that were made throughout the peer-review process. We cite this analysis plan in our methods section, stating: “An analysis plan was prepared in April 2020, which we have updated to reflect changes made throughout the peer review process (see Analysis Plan).” (Page 5, Lines 3–5)

**ACADEMIC EDITOR COMMENTS**

**1.** Why is this analysis conducted using person-months rather than person-days? These are insurance claim data, so presumably the authors have access to exact dates. They could then revise the exposure variable to be = 1 if the temperature within a time window that is much smaller (e.g., daily temperature, or average daily temperature within a 3-day window, etc.) than the time window used in the present analysis (monthly) exceeds the long-term average by 1.5C. R2's comment about lagged effect is relevant here.

We thank the Editor for this important question. We discussed the data structure extensively when we initially conceived of the study, in line with previous literature.[1-3] In revisiting the question of person-time for this revision, we still feel that the person-month data structure is most appropriate for the present study for the following three reasons:

First, we anticipate that individual- and hospital-level differences in timing and delays to treatment seeking would introduce considerable noise in the time elapsed between exposure to anomaly and the resultant healthcare visits. While there are several prior examples in the environmental health literature that use dates of hospitalization to examine the effects of environmental exposure (e.g., air pollution) on acute-onset health outcomes (e.g., myocardial infarction [MI]), the onset of neurologic symptoms in response to increased core temperatures often occurs gradually. Therefore, biologically, a person-day analysis is less appropriate for MS exacerbations than for very acute conditions, such as MI. Patients with MS are also typically advised to wait *at least* 24 hours after symptom onset prior to presentation [4, 5], and moreover in certain locations patients with MS may receive immediate care, while in other locations there may be considerable delays before a provider sees them. Second, our current approach minimizes outcome misclassification. Person-time is accrued based on insurance eligibility, which is measured by calendar months. Therefore, the use of person-months allows for a clearly and precisely defined denominator throughout the study period, whereas the creation of smaller intervals (i.e., weeks or days) would introduce person-time with partial insurance (and therefore outcome) eligibility.

Finally, and perhaps most importantly, we feel that the data structure is best motivated by our research question. We have now articulated more clearly in the introduction that: “We identify anomalously warm weather at the county level based on the extent to which monthly temperatures deviate from the long-term average temperature. This measure is intended to capture indolent changes in seasonal weather patterns that are not necessarily recognizable as periods of extreme heat, occur year-round, and may have health implications for certain groups, including individuals with MS.” (Page 4, Lines 37–41)

**2.** Related to the above, why is the exposure dichotomized? Is there reason to expect that a greater number of _days_ of exposure to anomalous weather could produce more exacerbations compared with fewer days of exposure? One could imagine an analysis in which the authors calculate anomalous weather in days -21 to -14, days -13 to -7, days -6 to -1, day 0, days 1-6, days 7-13, days 14-21, etc. (For the post-exposure bins, please see my comment below about placebo exposure.) Alternatively, is there any reason to expect that greater exposure to anomalous weather would lead to more severe exacerbations (e.g. longer hospital stays)? Or, is there reason to expect that larger deviations from average weather in warmer regions/seasons might lead to more severe exacerbations? Some of the examples in the text (e.g., 2003 French heat wave) are true "heat waves" -- when the temperature spikes to 98 in a Chicago summer [+15 deg compared with the monthly average] one would expect to see many more MS exacerbations compared with days when the temperature spikes to 65 in a Boston November [+15 deg compared with the monthly average]).

(The authors have used a number of different thresholds for temperature difference, e.g. +0.5, +1.0, +2.0 deg, but why not model exposure as a categorical variable or using a spline, rather than as binary?)

The point the Editor makes about severity is an interesting one. Because duration of hospitalization reflects a number of factors unrelated to disease severity – including patient preference, provider preference, the patient’s degree of disability and comorbid disease, factors relating to housing and placement, and the insurance provider’s willingness to pay for an extended hospitalization – such an analysis would be informative in a data source where more direct measures of disability are available (i.e. EDSS, which is available in the Danish MS registry data) or in electronic health record (EHR) data where relatively more details regarding the hospitalization are available.

In response to this comment, we have, however, added a number of additional sensitivity analyses. First, we created two alternative definitions of our main exposure: First, we created a categorical variable for the magnitude of the deviation between monthly average and long-term average exposures. Here, the referent category includes months in with the monthly average temperature was equal to or less than the long-term average. We find that the greater the magnitude of the deviation of the monthly average temperature from the long-term average, the stronger the association with MS-related healthcare visits. This was particularly true when the deviation exceeded 4.5˚C for emergency visits (RR=1.056, 95% CI: 0.998–1.117) and inpatient hospital visits (RR=1.119, 95% CI: 1.070–1.171). (Supplemental Table 9)

Second, we calculated the number of days per month that deviated from the long-term average for that calendar day by at least 1.5˚C and created a continuous and categorical version of this variable with zero anomalous days as a referent. We also find stronger associations when anomalous days were more frequent. Specifically, we find that during months in which there were more than fourteen anomalously warm days, the risk was increased for both emergency visits (RR=1.052, 95% CI: 0.990 – 1.117) and inpatient hospitalizations (RR=1.070, 95% CI: 1.024–1.117) (Supplemental Table 12)

Collectively, the above results are consistent with the original 1.5˚C threshold analysis, which was selected in line with the Paris Agreement. Collectively, they imply that greater deviations in temperature are associated with greater risk for acute care visits. We have noted in this in the discussion section, saying: “Sensitivity analyses further suggested that greater deviations in monthly average temperature from the long-term average conferred greater risk for treatment seeking related to MS in the acute care setting.” (Page 9, Lines 6–7)

Finally, in response to this comment and comments from Reviewer 2, we conducted a lagged analysis, in which we examined the association between MS-related healthcare visits and anomalies lagged one month into the past and one month into the future. We find evidence of a weak association between MS-related inpatient visits and anomalies during the previous month (RR=1.028, 95% CI: 1.010–1.047). However, as expected, we observed no evidence that anomalously warm weather one month in the future increased risk for MS-related healthcare visits. (Supplemental Table 12)

3. Why is the baseline reference identified as the "long-term average for that month and county over the study period"? If the hypothesized mechanism is that MS exacerbations are due to "sudden increases in ambient or core body temperature", wouldn't a more appropriate referent be something like the average temperature over the prior month?

Because the present analysis uses administrative claims data, we use MS-related healthcare visits as an indirect proxy measure for the recrudescence of neurologic symptoms in response to increases in core body temperature. We have attempted to more clearly articulate our assumed underlying mechanism in the introduction section by stating: “We hypothesized that during periods of anomalously warm weather, individuals with MS would be more likely to experience neurological symptoms, resulting from either physiological or behavioral changes, that prompt treatment-seeking.” (Page 4, Lines 41–43) We further elaborate in the discussion section, stating: “In warmer seasons, such as spring or summer, anomalously warm weather could plausibly lead to increases in core body temperature sufficient to increase risk of MS admission via additional heat stress. During anomalously warm winter or fall months, modified behavior, such as increased outdoor physical outdoor exertion, could also lead to increases in core body temperature.” (Page 9, Lines 20–24)

In response to the Editor’s suggestion that the previous month is a more appropriate baseline than the long-term average, we created an alternative exposure metric that corresponds to the deviation of monthly average temperatures from the previous months. The results are presented below, but this metric is highly correlated with calendar month – much more so than our originally defined anomaly metric – because temperatures generally increase as compared with the previous month in February–July and decrease as compared with the previous month in September–January as depicted in the figure below making it difficult to disentangle the effects of seasonality versus increasing average temperatures in this analysis. For this reason, we propose not to include the analysis in the revised manuscript.

|  | **Overall**  RR (95% CI) | **Women**  RR (95% CI) | **Men**  RR (95% CI) |
| --- | --- | --- | --- |
| **Outpatient Visits ^5^** | 1.021 (1.017 – 1.024) | 1.023 (1.019 – 1.027) | 1.014 (1.008 – 1.019) |
| **Emergency Visits** | 1.004 (0.992 – 1.018) | 1.002 (0.987 – 1.017) | 1.011 (0.988 – 1.034) |
| **Inpatient Visits** | 1.018 (1.008 – 1.028) | 1.020 (1.008 – 1.032) | 1.011 (0.988 – 1.034) |

**4.** I appreciate that the authors have estimated region- and season-specific models. But on this point I am in agreement with R2 who asks: “Is an excess of 1.5 degrees in winter really heat stress?” It seems reasonable to hypothesize that warm weather anomalies might produce exacerbations during warm weather months (e.g., when the temperature is 100 degrees instead of 95 degrees as expected), but is it reasonable to expect a symmetric effect during cold weather months? (Do hospitals in Boston experience an increase in acute MS care utilization when the temperature hits 70 degrees in November?) I am surprised that the strongest associations were observed in the winter months. Perhaps the manuscript would benefit from additional detail.

In response to this comment from the Editor and to comments from Reviewer 2, we have endeavored to clarify (1) the framing of our research question; (2) the presumed mechanism underlying the association between anomalies and acute care visits, and (3) why anomalies in the winter could plausibly lead to increases in core body temperature.

First, with respect to the framing of our research question: because is well-established that the majority of individuals with living with MS are sensitive to extreme heat, our objective was not to redemonstrate heat sensitivity in patients with MS at the population level. Rather, the major contribution of this study is our examination of whether individuals living with MS are sensitive to temperatures that deviate above the long-term norm to which populations are otherwise acclimatized – circumstances that are expected to become increasingly frequent as the global climate changes. We have revised the introduction to more clearly articulate our research question, stating that our measure of anomalies is “…intended to capture indolent changes in seasonal weather patterns that are not necessarily recognizable as periods of extreme heat, occur year-round, and may have health implications for certain groups, including individuals with MS.” (Page 4, Lines 39–41)

Second, we now explain the presumed underlying mechanism linking anomalies with MS-related healthcare visits in the introduction stating, “We hypothesized that during periods of anomalously warm weather, individuals with MS would be more likely to experience neurological symptoms, resulting from either physiological or behavioral changes, that prompt treatment-seeking.” (Page 4, Lines 41–43) Finally, we specifically address how exposure to higher-than-average temperatures could plausibly lead to increases in core body temperature year-round in the discussion, stating: “It is therefore possible that while individuals with MS are able to successfully avoid exposure to high absolute temperature if they are behaviorally and physiologically adapted, they are relatively more susceptible to warmer-than-expected temperatures at all times in the year. In warmer seasons, such as spring or summer, anomalously warm weather could plausibly lead to increases in core body temperature sufficient to increase risk of MS admission via additional heat stress. During anomalously warm winter or fall months, modified behavior, such as increased outdoor physical outdoor exertion, could also lead to increases in core body temperature.” **(**Page 9, Lines 18–24)

**5.** I am in agreement with R2 that the negative control/falsification analysis -- perhaps it would be reasonable to cite either Lipsitch et al. (Epidemiol 2010;21:383-388) or Prasad & Jena (JAMA 2013;309:241-242) -- does not seem to be providing the hoped-for reassurance. In addition to the analysis presented, might the authors consider some type of placebo exposure where they estimate the effect of weather anomalies on MS utilization prior to the exposure period? (Logically, if the hypothesized mechanism is true, the weather anomaly cannot cause MS exacerbations _prior_ to the weather anomaly.)

In response to comments from Reviewer 2 and the Editor, we have provided some interpretation of our negative outcome control analysis. We anticipate that our findings could be explained by the fact that (1) individuals with MS may have other conditions that increase their sensitivity to temperature anomalies, or (2) some visits related to MS may not be coded as such. We have included a discussion of this analysis in our limitations section, saying: “In our negative outcome control analysis, we observe consistently attenuated but persistent associations between anomalously warm weather and MS-related visits. We anticipate that these residual associations reflect some combination of the fact that individuals with MS may have other conditions that increase their sensitivity to temperature anomalies (e.g., cardiovascular disease), and that some of these visits may truly have been related to MS but not coded as such.” (Page 10, Lines 42–46)

We have also incorporated two negative exposure control analyses as additional robustness checks. First, upon further consideration we feel that our secondary analysis of anomalously cool temperatures serves as a negative exposure control because there is no reason to anticipate that individuals with MS should experience symptom recrudescence with exposure to colder than anticipated weather. Second, we conducted a negative exposure control analysis in which we created a random permutation of the exposure variable within individuals and repeated our main analysis. In both of these analyses, we observe no evidence that supports an association between the negative exposure control and MS-related healthcare visits. (See Table 2 & Table 3)

As discussed above, our lagged analysis is reassuring insofar as we observed no evidence that anomalously warm weather one month in the future increased risk for MS-related healthcare visits.

**6.** Is it standard in the field to accept all visits flagged with ICD9=340 as being "multiple sclerosis related"? I would like to see a little more specificity here (e.g., "all visits with primary diagnosis ICD9=340 _and_ procedure code = 99.23 ("injection of steroid") (e.g., Laura A et al. Environ Res. 2016;145:68–73). In any case, I would expect the authors to revise the outpatient visits outcome variable along these lines (e.g., either ICD9=340 as a primary _or_ secondary diagnosis _and_ a steroid claim within 7 days -- see Ollendorf DA et al. J Manag Care Pharm 2002;8:469-76; and Chastek BJ et al. J Med Econ 2010;13:618-625).

We agree with the Editor that a more specific outcome definition is reasonable here. For this revision, we have restricted our outcome definition to only the first, second, and third diagnostic position in an attempt to balance sensitivity against specificity. These results are essentially the same as those presented in the original submission. We have not incorporated steroid claims into our outcome definition because we are not attempting to capture hospital admissions for true disease flares only. As we note in the introduction, the neurologic symptoms individuals with MS experience in response to increased core body temperatures are generally referred to as “pseudo flares” that do not represent underlying progression of the disease. While steroids are the standard of care for true relapse, pseudo-flares are typically treated with symptomatic management only [6].

**Minor comments:**

7. Was seasonality ("winter", "spring", etc) specific to latitude or altitude? (e.g., December in San Francisco is very different from December in Boston; and December in Gallup, New Mexico is very different from December in Phoenix, Arizona; and temperature spikes of +1.5 deg are much more manageable in Phoenix, where everyone has air conditioning, compared with in Boston or San Francisco, where many people do not have air conditioning)

Seasonality was not defined specific to latitude or altitude. We attempted to capture some of the heterogeneity the Editor refers to above by incorporate subgroup analysis by season, region, and by season within U.S. Census region. Moreover, all subgroup analyses incorporate state fixed effect. We also note in the discussion section that the lack of data on air conditioning use – and other measures of socioeconomic status – at the county level is a limitation of the present study. We state: “As a final limitation, these study data lack a number of important individual-level covariates. Namely, individual-level measures of socioeconomic status such as occupational status, household income, access to air conditioning, housing quality, and race/ethnicity may determine where individuals are located – and therefore the extent of their exposure to anomalously warm weather – and their propensity to seek treatment for neurologic symptoms.” (Page 10, Line 49–Page 11, Line 2)

In response to this comment and comments from Reviewer 2, we have also added an additional analysis by climate zones as defined by the U.S. Department of Energy. This analysis separately examines the association between anomalies and MS-related healthcare visits among individuals in subarctic, very cold, cold, mixed-humid, mixed-dry, hot-humid, hot-dry, and marine climates. Climate zone was assigned at the county-level based on U.S. Department of Energy designations. Our results suggest that humidity may play a role in the observed relationship between anomalies and MS-related visits: “By climate zones, we observed stronger associations in cold and humid climates and weaker or null associations in dry and marine climates. Individuals living in hot-humid climates had the strongest associations for both emergency department visits (RR=1.109, 95% CI=1.053–1.168) and inpatient visits (RR = 1.090, 95% CI: 1.048–1.135).” (Page 8, Lines 5–8) **8.** In the negative control analysis, the authors specify that MS-unrelated visits were "any visit without diagnostic codes 340 or G35"-- are they referring to just the primary diagnosis or also the secondary diagnosis?

Visits unrelated to MS were those without diagnostic codes 340 or G35 in any diagnostic position (one through 25). In the methods section, we now state: “We created an indicator variable for visits *unrelated* to MS which equaled one for outpatient, emergency, or inpatient visit in which the diagnostic codes 340 and G35 were absent from all diagnostic positions.” (Page 6, Lines 2–4)

**9.** Was the exposure adjusted for humidity/heat index? If not, why not?

We do not adjust for humidity or heat index, which is often highly colinear with temperature anomalies and, which we believe is more likely to modify than it is to confound the effect of anomalies on health outcomes. However, our secondary analysis by climate zone presents separate results for individuals living in Mixed-Humid and Hot-Humid climates (see response to comment 7 above).

**10.** Please consider shifting some of the supplemental tables to the primary text. Some of the supplemental tables are appropriately left in the appendix. But some of the supplemental tables address (or hope to address) aspects of the Bradford Hill criteria, so one might hope that these would be shifted to the main text.

In response to the Editor’s recommendations, we have incorporated two new negative exposure control analyses into the main text (see Tables 2 and 3). We are happy to incorporate any other results into the main text that the editor would like.

**Summary:**

This is an interesting manuscript, but given the relatively small magnitude effect sizes (and relatively small e-value), I need more convincing that the estimated effect is not simply a byproduct of the sample size.

We thank the Editor for their interest in our manuscript. We have attempted to strengthen the revised version by incorporating a number of additional secondary and sensitivity analyses, which we hope will provide a more comprehensive picture of the association between anomalies and MS-related healthcare visits. We also note that although the magnitude of the associations estimated herein are relatively small, they are (1) consistent with the magnitude of associations typically reported for environmental exposures (e.g., Shah et al. [7] find RR = 1.014 for mortality per 10 ppb); and (2) can affect the entire population simultaneously and thus have a large population health effect even with a relatively low RR.

**REVIEWER 1**

Review of the original article" Anomalously warm weather and acute care visits in patients with multiple sclerosis: A retrospective study of privately insured individuals in the U.S"

The paper sets out to investigate the effect of heat on acute care visits in patients with multiple sclerosis. The topic is timely is timely and interesting and the investigation of patients suffering from disorders in the central nervous system is needed. The authors have performed sound analyses complemented with relevant secondary and sensitivity analyses. However, some concerns need to be addressed before this study can be considered for publication.

**Major comments:**

Does the claims data set include date of death?

The version of the Optum data used in this analysis does not include date of death. Optum makes different versions of the data available for research purposes. Optum ZIP5 contains time-varying, five-digit zip codes but no date of death, and Optum DOD contains date of death and state only. Date of death and five-digit zip code cannot be linked at the individual level because of the risk of identifiability. Because our analysis is dependent on detailed geographic data for exposure assignment, we used Optum ZIP5 rather than Optum DOD for this study.

What happened if an individual changed state of residence or changed insurance?

The follow-up period should be described in more detail.

All location variables (county, state, region) are time-varying in the present study. Therefore, if an individual changed state this would be captured by a change in the value of the county and state variables. We clarify this in the methods section by stating: “Using five-digit ZIP codes from eligibility files, we assigned time-varying county, state, and region of residence (Northeast, South, Midwest, and West) for each respondent.” (Page 6, Lines 7–9)

The Optum database is comprised of individuals covered by a single, private U.S. insurance provider. We included all person-time in which an individual was covered by this insurance provider. If they changed insurance but later returned to their original insurance provider within the study period, this is captured with non-consecutive person-months of follow-up. Follow-up ended if an individual changed to another insurance provider and did not return within the study period. In response to the above comments, we have included the following text in the methods section: “Using insurance eligibility files, we created a person-month dataset in which follow-up extended from the first month in which an individual satisfied our inclusion criteria (i.e., the month in which their third MS-related claim occurred) until the last month of insurance eligibility or the end of the study period on December 31, 2017. In this open cohort, we defined eligible person-months based on insurance eligibility and excluded all person-months of follow-up in which an individual was not eligible for insurance. We made no other restrictions or exclusions based on duration of insurance eligibility.” (Page 5, Lines 18–23)

**4.** Even though the authors divide US into regions to investigate how an increase/decrease in temperature impacts care visits, and the cut-off of 1.5C is sound in a climate change perspective, a country wide study would benefit from adding some information on where this change is on the relative scale. That is, an increase of 1.5 C, may not be the same on the relative scale, even within region, which then would have implications on interpreting the results as one may be comparing a more "extreme" increase to one that is less so.

We agree with the reviewer, and to help better contextualize the relative meaning of a 1.5˚C increase in temperature we have included a supplemental table that presents the median, IQR, and min/max temperature for each region, and by season within each region. We note in the results section that “By region, on average, temperatures were highest in the South and lowest in the Northeast with greatest year-round variation observed in the Midwest and the least year-round variation in the West.” (Page 7, Lines 28–30) Further, we note in the discussion section that “The associations between warm weather and MS-related hospital visits also demonstrated substantial variation by season, region, and climate zone. By season, we observed the strongest associations during the winter. By region, we observed the strongest associations in the South and the weakest associations in the West, where temperatures exhibited the least year-round variability and where a 1.5˚C deviation from the long-term average therefore reflects the greatest relative deviation. These findings may be partially explained by climate.” (Page 9, Lines 41–46)

**Minor comments:**

The references 2-6 used to support that morbidity have been attributed to extreme heat previously are not attribution studies per se.

We now state in the introduction that “Morbidity and mortality due to heat exhaustion and heat stroke; exacerbation of underlying cardiorespiratory and other chronic diseases; and suicide and injury-related deaths have all been *associated with* high temperatures” to avoid the potentially erroneous implication that these are attribution studies. (Page 4, Lines 21–23)

Usually a 30-year window is used in climate studies. What would be the long term county average using 30-year windows instead of using the average over the study period and how would this affect the results?

Because temperatures have increased over the past three decades, we anticipate long-term county averages calculated over the study period will be higher than long-term county averages calculated using a 30-year window. We anticipate that use of the study period to calculate long-term averages is therefore conservative because it could lead us to underestimate the frequency of anomalies. Our approach is consistent with several recent studies that use the study period to calculate anomalies.[1, 3, 8, 9]

**Other:**

Present the RR and 95% CI in a consistent manner. Now not always the case.

The RR and 95% CI are now presented in a consistent manner throughout the results section.

**REVIEWER 2**

This paper uses routine data–sets to examine the relationship between temperature anomalies and multiple sclerosis health care utilisation. I was asked for a statistical report and I interpret that to include all aspects of the design and conduct of the study.

**Points of detail:**

Page 6 Why model age as categorical? Categorising an essentially continuous variable wastes information (Altman and Royston, 2006; Royston et al., 2006) and leads to models which are often implausible as they predict the effect remaining flat within categories and then jumping to a new value at the category boundary. In Figure S3 the authors use a spline so why not do that for age?

We agree with the Reviewer, and we have repeated all statistical analysis with age defined as a continuous – rather than categorical – variable with natural splines in all analyses that adjust for age.

**Pages 6 and 7.** The description of the analysis is rather brief and I do not think I could replicate it if the authors gave me the data. To be specific let me outline what I understand from the text so the authors can either confirm it or expand to dispel my confusion.

- The unit of analysis is the calendar month.
- Each month has an outcome value for each type of MS health usage. Is that binary or a count?
- If it is binary is this a log–binomial model (in R–speak family = binomial, link = log) or have the authors used what people have come to call a modified Poisson model as an approximation to the log–binomial to estimate risk ratios?
- Calendar year is presumably included as a fixed effect but is state a random effect given there are 48 of them?
- What happens if people move during their observation period? Is their location changed or are they treated as being where they started from?

We have expanded our description of the analysis in response to the points above:

- The reviewer is correct that the unit of analysis is the calendar month, and in the “Statistical Analysis” section, we now state: “We examined the association between anomalously warm weather and MS-related healthcare visit in an individual-level repeated measures framework. For all described analyses, the unit of analysis was the person-month.” (Page 6, Lines 15–17)
- The outcome is a binary variable that equals one if there was *any* MS-related healthcare visit and zero otherwise. We defined the outcome as a binary variable rather than a count because monthly claims exhibit significant overdispersion due to both a point mass at zero and right skew (in these data, the number of unique MS-related claims per month ranges from zero to 291). In the methods section, we state: “Because monthly claims exhibited overdispersion due to a point mass at zero and right skew, we created an indicator variable for each visit type that equaled one for any month in which an individual had at least one MS-related outpatient, emergency, or inpatient visit – respectively – and equaled zero otherwise.” (Page 5, Line 49–Page 6, Line 2)
- The former is correct – we used a binomial distribution and log-link to estimate risk ratios rather than odds ratios. We have clarified our methods, stating: “We used generalized linear models with the binomial family and log-link to estimate risk ratios (RR) for the association between anomalously warm weather and MS-related outpatient, emergency department, and inpatient visits, respectively.” (Page 6, Lines 21–23)
- Both calendar year and state are included as fixed effects in the present study. We chose fixed rather than random effects for state because of additional parametric assumptions implied by random effects – namely that states are drawn from a normal distribution u_j_ ~ i.i.d. N(0, σ_u_^2^ ). We now state in the methods section that we adjusted for covariates in all models, including: “…fixed effects for U.S. state and calendar year to account for regional differences in provider networks and secular trends, respectively.” (Page 6, Lines 25–26)
- All location variables (county, state, region) are time-varying in our analysis. We now state in the methods section that: “Using insurance eligibility files, we determined sex (female or male), age, and calendar year. Using five-digit ZIP codes from eligibility files, we assigned time-varying county, state, and region of residence (Northeast, South, Midwest, and West) for each respondent.” (Page 6, Lines 7–9)

Page 9 Are the sub–group differences presented here backed up by a formal test? If so it should be presented and if not then can we rely on these differences?

The subgroup analyses presented in this paper were specified *a priori* and the discussion of differences between subgroups included in the results section is qualitative. We have included – for readers who are interested – a test of formal statistical interaction for our subgroup analyses by sex, age, season, region, and climate zone is presented as a footnote in Supplemental Tables 2, 4-6, and 8. We note in the results section under the “Secondary Analyses” section subheading: “In formal tests of statistical interaction, we observed no statistically significant interaction by sex, age group, or season, but found statistically significant interaction by region and climate zone (Supplemental Materials).” (Page 8, Lines 8–10)

Page 22, Table Why are there so few from the north east?

This study population is comprised of individuals covered by a single, private U.S. insurer. The geographic distribution of study population reflects the size of the insured population in each region. We have noted in the limitations section that “nearly half of the study population resided in the South with relatively few individuals from the Northeast included (approximately 4% of the study population). Moreover, given the above noted regional differences and the unique nature of the U.S. healthcare system, our findings may not generalize to other geographic contexts.” (Page 10, Lines 33-36)

Page 23, Figure 2 The results for women seem much less precise than I would have expected given that they represent the majority of the sample. Is there any explanation for this?

We thank the reviewer for noting this error in Figure 2. We now present the correct confidence bands for the sex-stratified analysis in Figure 2.

**Points of more substance:**

I have some problems reconciling the science and the statistical analysis used here. I am certainly not an expert on the science here but let me express my doubts so the authors can consider them and if necessary rebut them.

**Heat stress:** On page 3 the authors state that ‘. . . heat sensitivity is a recognized feature of multiple sclerosis . . . ’. Their chosen exposure is heat anomalies and empirically (Figure S2) they found that in the United States these occur most often in the winter and that is when they found a relationship between exposure and outcome for at least some of their three outcomes (Table S5). Is an excess of 1.5° in winter really heat stress? At the time of writing my region is experiencing temperatures which are apparently above the seasonal average but at 14° I am not feeling any stress, unlike in the summer when they were in the high twenties, and nor do I think I am experiencing ”... core temperature–induced changes. . . ” (page 11). There is also the issue of the failure to find any relationship between average temperature and events as exemplified in Figure S3 which is more or less flat from -3° to +27°.

We thank Reviewer 2 for this important question. In response we have attempted to clarify key differences between anomalies and heat stress and have re-focused the introduction and discussion to better articulate the presumed mechanism by which our exposure of interest affects the outcome.

First, although much of the literature on the health effects of temperature is focused on extreme heat, this is not the focus of our study. Because is well-established that the majority of individuals with living with MS are sensitive to extreme heat, our objective was not to redemonstrate heat sensitivity in patients with MS at the population level. Rather, the major contribution of this study is our examination of whether individuals living with MS are sensitive to temperatures that deviate above the long-term norm to which populations are otherwise acclimatized – circumstances that are expected to become increasingly frequent as the global climate changes. We have revised the introduction to more clearly articulate our research question, stating that our measure of anomalies is “…intended to capture indolent changes in seasonal weather patterns that are not necessarily recognizable as periods of extreme heat, occur year-round, and may have health implications for certain groups, including individuals with MS.” (Page 4, Lines 39–41)

Further, we agree with Reviewer 2 that mere exposure to temperatures that deviate 1.5˚C in winter does not qualify as heat stress. However, exposure to temperatures that are higher than expected could plausibly lead to increases in core body temperature – even in the winter – for example via physical exertion or if individuals wear extra layers in anticipation of cooler temperatures. We discuss this, along with the apparent discrepancy between the observed association with anomalies and the lack of association with absolute temperature in the discussion section: “Because heat sensitivity is a known clinical feature of MS, patients are often counseled to avoid the provocative effects of extreme heat and are therefore more likely to take precautions against high absolute ambient temperatures [22]. It is therefore possible that while individuals with MS are able to successfully avoid exposure to high absolute temperature if they are behaviorally and physiologically adapted, they are relatively more susceptible to warmer-than-expected temperatures at all times in the year. In warmer seasons, such as spring or summer, anomalously warm weather could plausibly lead to increases in core body temperature sufficient to increase risk of MS admission via additional heat stress. During anomalously warm winter or fall months, modified behavior, such as increased outdoor physical outdoor exertion, could also lead to increases in core body temperature. Collectively, our results therefore suggest that patients with MS may need to work with their healthcare providers to anticipate and protect themselves from warmer-than-expected temperatures even when absolute temperatures are more moderate or even cool.” (Page 9, Lines 15–27)

**Role of negative control:** The authors state that the relationship between temperature anomalies and non–MS related events was attenuated. Given the considerable overlap between the intervals I am not convinced that the negative control shows what the authors hoped. Clearly something is going on here but it seems complex.

We agree with the reviewer that the negative outcome control presented with our original submission implies some relationship between anomalies and healthcare visits unrelated to MS. We anticipate that this could be explained by the fact that (1) individuals with MS may have other conditions that increase their sensitivity to temperature anomalies, or (2) some visits related to MS may not be coded as such. We have included a discussion of this analysis in our limitations section, saying: “In our negative outcome control analysis, we observe consistently attenuated but persistent associations between anomalously warm weather and MS-related visits. We anticipate that these residual associations reflect some combination of the fact that individuals with MS may have other conditions that increase their sensitivity to temperature anomalies (e.g., cardiovascular disease), and that some of these visits may truly have been related to MS but not coded as such.” (Page 10, Lines 42 – 46)

We have also incorporated two negative exposure control analyses as additional robustness checks. First, upon further consideration we feel that our secondary analysis of anomalously cool temperatures serves as a negative exposure control because there is no reason to anticipate that individuals with MS should experience symptom recrudescence with exposure to colder than anticipated weather. Second, we conducted a negative exposure control analysis in which we created a random permutation of the exposure variable within individuals and repeated our main analysis. In both of these analyses, we observe no evidence that supports an association between the negative exposure control and MS-related healthcare visits. (See Table 2 & Table 3)

**Leads and lags:** Working with existing data–sets one has to take what one can find but the data available to the authors here is best suited if the relationship between anomaly and event is close in time. If heating takes a time to have its effect then events may depend more on the heat over the previous week or two and that may well have been in the previous month.

In response to this comment, we have included an analysis in which the exposure is lagged backward one month and forward one month. The strongest associations between anomalies and MS-related healthcare visits occur in the same month, although we observe some evidence of a weak association between MS-related healthcare visits and anomalies that occurred during the previous month. Our findings presented in Supplemental Table 12 and are summarized in the results section: “In our analysis with the lagged exposure variable, we found that anomalously warm weather during the previous month was weakly associated with MS-related inpatient visits (RR=1.028, 95% CI: 1.010–1.047). However, we observed no evidence that anomalously warm weather one month in the future increased risk for MS-related healthcare visits.” (Page 8, Lines 25–28)

**Generalisability:** Will this apply to regions of the world with different climates? Specifically, will it apply to a region like Europe some of which is in similar latitudes but has a different climate? There are classifications of climate and I wonder whether it might be more generalisable to use climate zones rather than the defined United States regions.

We have also added an additional analysis by climate zones as defined by the U.S. Department of Energy. This analysis separately examines the association between anomalies and MS-related healthcare visits among individuals in subarctic, very cold, cold, mixed-humid, mixed-dry, hot-humid, hot-dry, and marine climates. Our results suggest that humidity may play an important role in the observed relationship between anomalies and MS-related visits: “By climate zones, we observed stronger associations in cold and humid climates and weaker or null associations in dry and marine climates. Individuals living in hot-humid climates had the strongest associations for both emergency department visits (RR=1.109, 95% CI=1.053–1.168) and inpatient visits (RR = 1.090, 95% CI: 1.048–1.135).” (Page 8, Lines 5–8)

**Summary:** A few points for clarification and some points about the analysis strategy.

We thank Reviewer 2 for their comments, and we have attempted to address them as fully as possible.

**REVIEWER 3**

This is an elegant study of the impact of temperature anomalies on MS-specific healthcare utilization making excellent use of extant big data.

The authors have been diligent in recognizing the inherent limitations in these data and have adopted appropriate methodological and analytical strategies to control for these limitations.

My only criticism, suggestions really, would be to modify the abstract to report the RR as opposed to the excess number of visits. Given the very small increased risk, reporting the excess number of visits overstates the magnitude of the observed effect. You could state in the abstract that the observed effect was increased when a 2.0C threshold was used to address magnitude of the effect.

Overall, this was a well written report of a complex study with a number of different analytical steps. Kudos.

We thank the Reviewer for these comments. We also agree that the results presented in the abstract are not representative of our primary analysis and exaggerate the magnitude of the observed effects. In response, we now present RRs in the “Methods and Findings” section of the abstract, stating: “Anomalously warm weather was associated with increased risk for emergency department visits (RR=1.043, 95% CI: 1.025–1.063) and inpatient visits (RR=1.032, 95% CI: 1.010–1.054). There was limited evidence of an association between anomalously warm weather and MS-related outpatient visits (RR=1.010, 95% CI: 1.005– 1.015).”

**References**

1.	Parks, R.M., et al., *Anomalously warm temperatures are associated with increased injury deaths.* Nature medicine, 2020. **26**(1): p. 65-70.

2.	Mares, D., *Climate change and crime: monthly temperature and precipitation anomalies and crime rates in St. Louis, MO 1990–2009.* Crime, law and social change, 2013. **59**(2): p. 185-208.

3.	Harp, R.D. and K.B. Karnauskas, *The influence of interannual climate variability on regional violent crime rates in the United States.* GeoHealth, 2018. **2**(11): p. 356-369.

4.	Lublin, F.D. and S.C. Reingold, *Defining the clinical course of multiple sclerosis: results of an international survey.* Neurology, 1996. **46**(4): p. 907-911.

5.	Schumacher, G.A., et al., *Problems of experimental trials of therapy in multiple sclerosis: report by the panel on the evaluation of experimental trials of therapy in multiple sclerosis.* Annals of the New York Academy of Sciences, 1965. **122**(1): p. 552-568.

6.	Frohman, T.C., et al., *Uhthoff's phenomena in MS—clinical features and pathophysiology.* Nature Reviews Neurology, 2013. **9**(9): p. 535-540.

7.	Shah, A.S., et al., *Short term exposure to air pollution and stroke: systematic review and meta-analysis.* bmj, 2015. **350**: p. h1295.

8.	Ruderman, D. and E.G. Cohn, *Predictive extrinsic factors in multiple victim shootings.* The Journal of Primary Prevention, 2020: p. 1-17.

9.	Kontis, V., et al., *Magnitude, demographics and dynamics of the effect of the first wave of the COVID-19 pandemic on all-cause mortality in 21 industrialized countries.* Nature medicine, 2020: p. 1-10.
